# The effect of facial expression on contrast sensitivity: A behavioural investigation and extension of Hedger, Adams & Garner (2015)

**DOI:** 10.1371/journal.pone.0205621

**Published:** 2019-11-06

**Authors:** Abigail L. M. Webb, Paul B. Hibbard

**Affiliations:** Department of Psychology, University of Essex, Colchester, United Kingdom; University of Leipzig, GERMANY

## Abstract

It has been argued that rapid visual processing for fearful face expressions is driven by the fact that effective contrast is higher in these faces compared to other expressions, when the contrast sensitivity function is taken into account. This proposal has been upheld by data from image analyses, but is yet to be tested at the behavioural level. The present study conducts a traditional contrast sensitivity task for face images of various facial expressions. Findings show that visual contrast thresholds do not differ for different facial expressions We re-conduct analysis of faces’ effective contrast, using the procedure developed by Hedger, Adams and Garner, and show that higher effective contrast in fearful face expressions relies on face images first being normalised for RMS contrast. When not normalised for RMS contrast, effective contrast in fear expressions is no different, or sometimes even lower, compared to other expressions. However, the effect of facial expression on detection in a backward masking study did not depend on the type of contrast normalisation used. These findings are discussed in relation to the implications of contrast normalisation on the salience of face expressions in behavioural and neurophysiological experiments, and also the extent that natural physical differences between facial stimuli are masked during stimulus standardisation and normalisation.

## Introduction

Fearful facial expressions are particularly salient to the human visual system, receiving preferential allocation of attentional resources, and inhibiting this attention from relocating to different stimuli [1–4]. This attentional effect is also found when fearful faces appear in peripheral vision [5–6]. When fearful expressions compete with salient noise stimuli for visual awareness, with the face and noise presented to different eyes, they break suppression faster compared to neutral faces [7–8], and are associated with increased activity in subcortical threat-processing regions even when observers report not having observed a face [5–6, 9]. These findings converge on the notion that the human visual system has evolved specific visual neural mechanisms that enable rapid identification of fearful expressions. This concept is reminiscent of LeDoux’s [10] ‘quick and dirty pathway’ for processing environmental information necessary for successful threat-avoidance.

A visual stimulus might be selectively processed for two reasons: because it is semantically and meaningfully relevant, or because its configuration is somehow congruent with low-level mechanisms in early vision that allow for it to be rapidly and efficiently processed. In terms of the threat bias for fearful faces, this means that fearful faces may be prioritised because of their emotional relevance, or their low-level image properties. The latter, low-level approach has been a particular focus within visual psychophysics, where studies have shown that it is specifically the low spatial frequency information in fearful faces that gives rise to the saliency effects associated with fearful expressions [1, 11–12]. Low frequency components of fear expressions are thought to undergo rapid processing via low-frequency-sensitive subcortical pathways that directly access the amygdala [11–12]. Such findings are interpreted as evidence of visual mechanisms that selectively respond to signals present in fearful faces [13–14]. Hedger, Adams and Garner [15] propose an equally low-level, but directionally different account, arguing that stimulus properties characteristic of fearful expressions ensure strong responses in the early stages of visual processing [7, 15]. Both Gray [7] and Hedger and colleagues [15] make use of this sensory bias hypothesis to explain how perceptual biases for fear expressions may be accounted for by the way in which their physical attributes are well-matched to the sensitivity of early visual processing, as opposed to the recruitment of attentional mechanisms that preferentially respond to expressions of fear [15, 16]. According to this notion, a distinction is made between stimulus detection that arises from attentional processes, and that which occurs pre-attentively. Here, Hedger and colleagues [15] implicate the contrast sensitivity function in the threat bias.

Hedger, Adams and Garner [15] compared the Fourier amplitude spectra of images of fearful and neutral faces, since both the overall contrast and the spatial frequency content of images are known to modulate stimulus salience, with the human visual system being most efficient at detecting information around 3–5 cycles per degree (cpd). They assessed the effective contrast of images by multiplying the Fourier amplitude spectra of face stimuli by a standard measure of the contrast sensitivity function, based on the Modelfest data set [17]. This approach quantifies effective contrast as the product of the image amplitude, and the visual system’s sensitivity, at each spatial frequency. They found that fearful faces, when matched for RMS contrast, were higher in effective contrast, and therefore better matched for the contrast sensitivity function compared to neutral faces. This finding was accounted for by the higher degree of contrast energy in midrange spatial frequencies, where the contrast sensitivity function peaks, in fearful faces. The effect was found to be consistent across several commonly used face databases including the Karolinska Directed Emotional Faces [18], Radboud Faces [19], Ekman and Friesen [20], Montreal set of facial displays (MSFDE) [21] and NimStim databases [22]. Data from their image analyses support the notion that biases for fearful expressions are driven at least in part by their sensory efficacy.

However, there remain several elements of this approach that are not addressed by Hedger, Adams and Garner [15]. The first relates to the behavioural evidence in support of the efficacy-account. Evidence for a role of preconscious processing of threat-information is provided by the results of experiments using the continuous flash suppression (CFS) paradigm, but does not directly measure expression-related effects on contrast sensitivity. That is, while the more rapid detection of fearful faces in CFS experiments is consistent with their greater effective contrast, we might also expect differences in contrast sensitivity to different facial expressions to be evident more directly. Specifically, if it is the case that the Fourier amplitude spectrum of fearful faces is well-matched to the human contrast sensitivity function, we should expect to observe an increase in contrast sensitivity, reflected by decreased contrast thresholds for fearful faces. The second issue is that prior to the transformation of face images, Hedger and colleagues [15] normalised them for their luminance and RMS contrast, such that they were identical on these measures at the physical level. While this is a commonly employed technique in psychophysical studies, performed to reduce contrast- and luminance-driven differences in stimulus salience, the process of attributing the aggregate physical contrast to all facial stimuli may mask naturally occurring differences in contrast between expressions in a way that could obscure results. Normalising images of natural scenes ensures a degree of consistency between images’ physical and perceived salience. However, the same may not be true when applied to face images. O’Hare & Hibbard [23] show inconsistencies between images’ physical and apparent contrast when there are differences in amplitude spectrum, when these stimuli are matched for RMS contrast. Given these uncertain effects of normalisation on the physical and perceived salience of facial stimuli, it is reasonable to question the degree to which normalisation influences results from both image analyses and behavioural paradigms. In particular, any consistent differences in RMS contrast across facial expressions would be expected either to increase, or cancel out, differences in sensitivity that can be attributed to differences in effective contrast.

To address these questions, we conducted a replication of the image analyses performed by Hedger, Adams and Garner [15]. We included face stimuli that are physically matched for RMS contrast, but also faces that were physically unmatched, such that they contain natural differences in both physical and apparent contrast. Furthermore, we conducted a traditional contrast sensitivity task in order to psychophysically test predictions from Hedger’s image analysis. We employed facial expressions as opposed to sinusoidal grating stimuli to measure expression-related differences in contrast sensitivity. An important feature of this latter study is that it directly addresses the association between face expression and contrast sensitivity at the behavioural level. In our final experiment, we measured the efficacy of meta-contrast masking for facial expressions either presented with their original contrast, or matched for physical contrast. This allowed us to determine the extent to which the effects of facial expression on stimulus detectability is influenced by normalising stimuli for RMS contrast.

## Experiment 1: Contrast sensitivity and image analyses

### Materials and methods

#### Participants

Eighteen (15 women, 3 men) participants took part in the study. All participants were informed of the nature of the study and provided written informed consent prior to the study beginning. The University of Essex Ethics Committee approved the employed experimental procedures. All participated in the experiment as part of a credited research module assessment, or in exchange for monetary reward. All participants had normal to corrected vision.

#### Stimuli and apparatus

Stimuli were grayscale images of 16 individuals, 8 males and 8 females, taken from the Karolinska Directed Emotional Faces set [18]. Face images were cropped to include internal features only, and included 4 emotional expressions of neutral, fear, anger and happiness. This allowed us to include positively and negatively-valenced comparisons in addition to fearful faces. All individual faces were presented in their normal, upright form, and in a phase scrambled format. Phase scrambled versions of the face images were used as a control measure, providing versions of faces whose configural content was disrupted but low level statistical properties preserved. Phase scrambling was performed using MATLAB fast Fourier transform functions. Contrast thresholds were determined using an adaptive staircase technique (see under Procedure, below). Stimuli were presented using a VIEWPIXX 3D monitor (52cm X 29cm), viewed from a distance of 65 cm. The stimulus size of faces was 5.5 degrees. The screen resolution was 1920x1080 pixels, with a refresh rate of 120Hz and an average luminance of 50 cdm^-2^. Each pixel subtended 1.43 arc min. Stimuli were presented at 10-bit resolution. Participants’ responses were recorded using the RESPONSEPixx response box. Stimuli were generated and presented using MATLAB and the Psychophysics Tool box extensions [24–26].

#### Procedure

Participants were tested individually in a quiet room and informed prior to the experiment that the study was concerned with face perception.

As a 2AFC location task, participants’ objective was to indicate, using 1 of 2 buttons on a RESPONSEPixx response box whether the target image (including faces and phase scrambled faces) appeared to the left or right of centre. The beginning of each trial commenced with the face stimulus on the left or right side of the screen. Participant responses determined the onset of the next trial. The proportion of times that the participant correctly indicated the location of the stimulus was recorded for all face stimuli.

The adaptive staircase method was used to establish the Michelson contrast required for correct detection (75% of the time) for each expression stimulus. The starting contrast level for each expression’s staircase began at 0.01 Michelson contrast. According to the up-down rule [27], Michelson contrast was increased by one initial step of 0.005 proceeding 1 incorrect observer response, thus boosting stimulus visibility. Conversely, 3 correct observer responses triggered a decrease in Michelson contrast, initially by 0.005. The overall staircase length was 70 trials, where the initial step size (0.005 Michelson) halved after 17, 35 and 52 trials. Four experimental blocks were completed, and the 280 trials for each combination of expression and phase scrambling were combined to create a single psychometric function.

## Results

### Contrast sensitivity data

**Fig 1:**
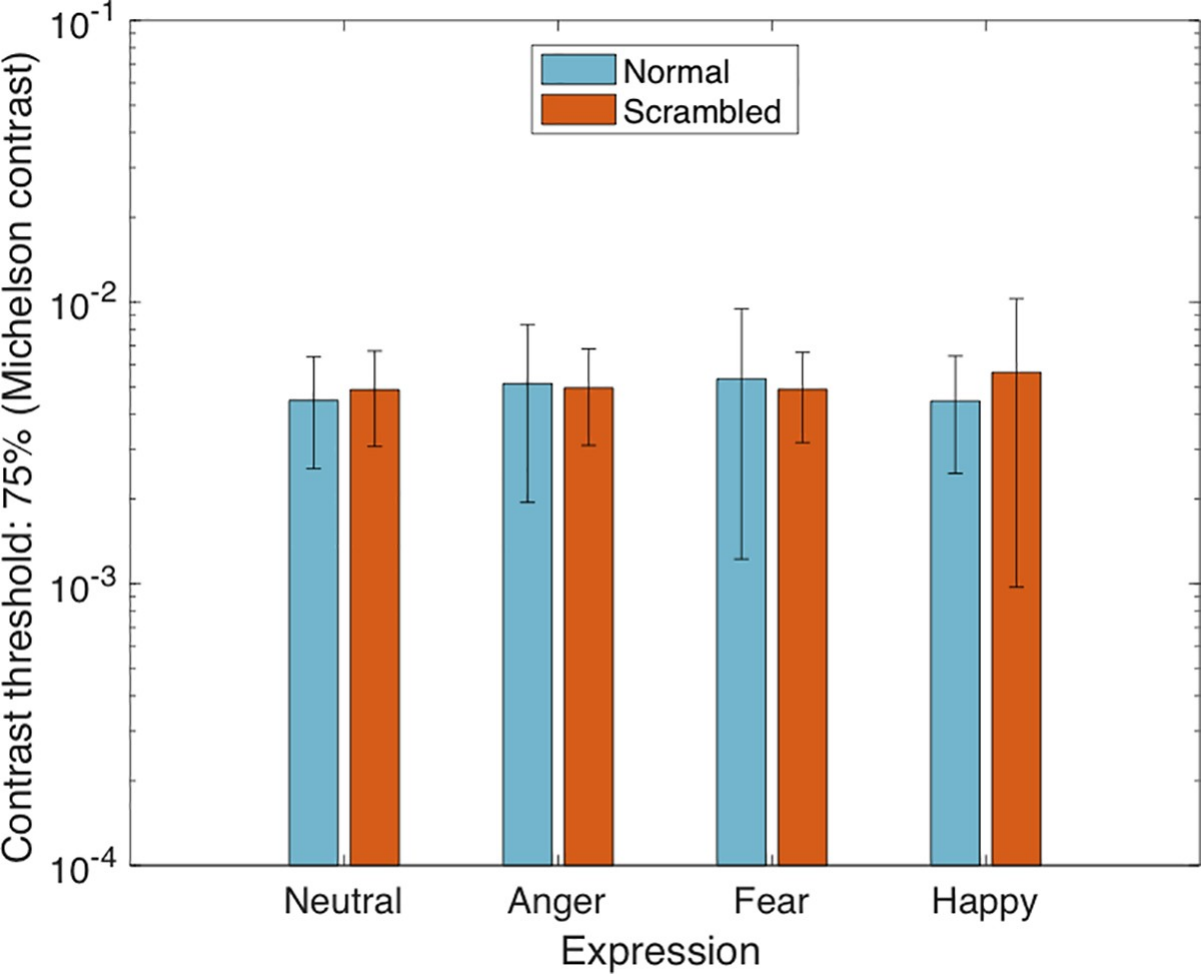
Visual contrast thresholds for face expressions. Thresholds for neutral, angry, fearful and happy facial expressions. Faces are unfiltered. Fearful face expressions are not associated with lower visual contrast thresholds, contrary to what might be predicted from Hedger and colleagues [15]. Error bars represent ±1 standard deviations.

The proportions of participants’ correct responses for each expression, at each contrast level, were used to create a psychometric function. A cumulative Gaussian function was fit to this data using the Palemedes toolbox [28] and used to determine a contrast detection threshold for each expression in its normal and manipulated (scrambled) formats. This 75% contrast detection threshold was defined as the contrast required for the participant to correctly identify the location of the face stimulus on 75% of trials. These results are plotted in Fig 1.

A 4 Emotion (neutral, anger, fear, happy) x 2 Manipulation (normal, scrambled) within subjects ANOVA revealed no significant effects of expression (*F*(3, 51) = .31, *p* = .82, ηp ^2^ = .018), or manipulation (*F*(1, 17) = .16, *p =* .70, ηp ^2^ = .009), and no significant expression x manipulation interaction (*F*(3, 51) = 1.14, *p =* .34, ηp ^2^ = .06). Analyses were repeated for contrast thresholds that were calculated using the RMS contrast of face stimuli. To do this, the RMS contrast of each stimulus was calculated (this scales linearly with changes in Michelson contrast) prior to the fitting of the psychometric function. A 4 Emotion (neutral, anger, fear, happy) x 2 Manipulation (normal, scrambled) within subjects ANOVA revealed no significant effect of expression (*F*(3, 51) = .42, *p* = .74, ηp ^2^ = .024), or manipulation (*F*(1, 17) = .07, *p =* .80 ηp ^2^ = .004), and no significant expression x manipulation interaction (*F*(3, 51) = 1.23, *p =* .31, ηp ^2^ = .07). These findings show that visual contrast thresholds do not vary between face expressions, nor are these findings different according to the two contrast metrics used here (Michelson and RMS). The absence of an expression-related effect on contrast sensitivity provides evidence against Hedger and colleagues’ [15] original claim that fear expressions (compared to neutral faces) exploit the contrast sensitivity function. In an attempt to understand the inconsistency between the present behavioural data, and that generated from image analyses by Hedger and colleagues [15], we conducted the same measure of faces’ effective contrast as that performed by Hedger, Adams and Garner [15] and extended this to include expressions of anger, happiness and disgust, including a condition where all face images had been either normalised for RMS contrast (as was the procedure for Hedger and colleagues [15]) or non-normalised, such that face images were analysed in their raw format, containing possible natural variations in RMS contrast.

### Image analyses

Hedger and colleagues [15] calculated the effective contrast for face images extracted from 5 face databases: NimStim, KDEF, Radboud, Montreal and Ekman and Friesen face sets. Stimuli were cropped to include internal features only and normalised for RMS contrast prior to analyses.

**Fig 2:**
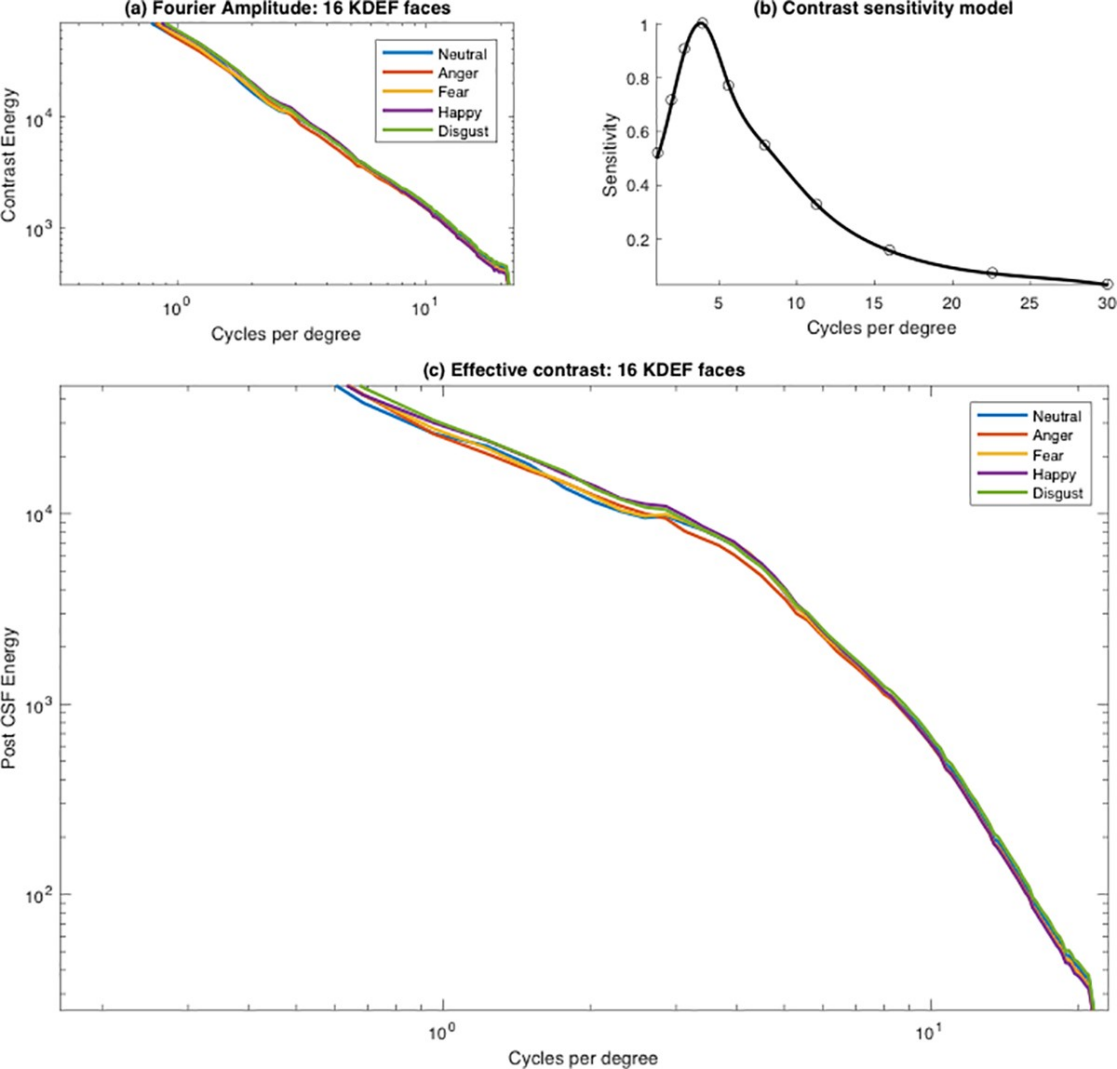
Calculating faces’ effective contrast. (A) The mean amplitude spectrum for each of the five expressions. (B) The contrast sensitivity function based on the ModelFest data. (C) The effective contrast, obtained by multiplying the original amplitude function by the contrast sensitivity function. This method for calculating effective contrast replicates that used by Hedger, Adams and Garner [15].

Effective contrast was calculated for the 16 KDEF face images used in our experimental study, referring to the same procedure described by Hedger, Adams and Garner [15]. First, Fourier amplitude spectra were calculated for each face image. We assumed a width for each cropped face of 7 degrees of visual angle, consistent with an assumed viewing distance of approximately 90 cm. From the ModelFest dataset [17], we extracted visual contrast thresholds for 10 stimulus parameters. These corresponded to Gabor stimuli, ranging from 1.12–30 cycles/degree. A smooth curve was fit to the average threshold (over 4 repetitions and all observers in the ModelFest dataset) using a cubic spline. The resulting contrast sensitivity function was then multiplied by the Fourier amplitude spectrum for each face image to establish each face’s effective contrast. Fig 2 shows an example of the procedure for calculating effective contrast for the 16 face images used in the present contrast sensitivity study. To extend our analysis, effective contrast was measured for face images across 4 of the face databases employed by Hedger, Adams and Garner [15], with the exception of the Ekman & Friesen face set [20]. The latter was not included as it is not freely available.

**Fig 3:**
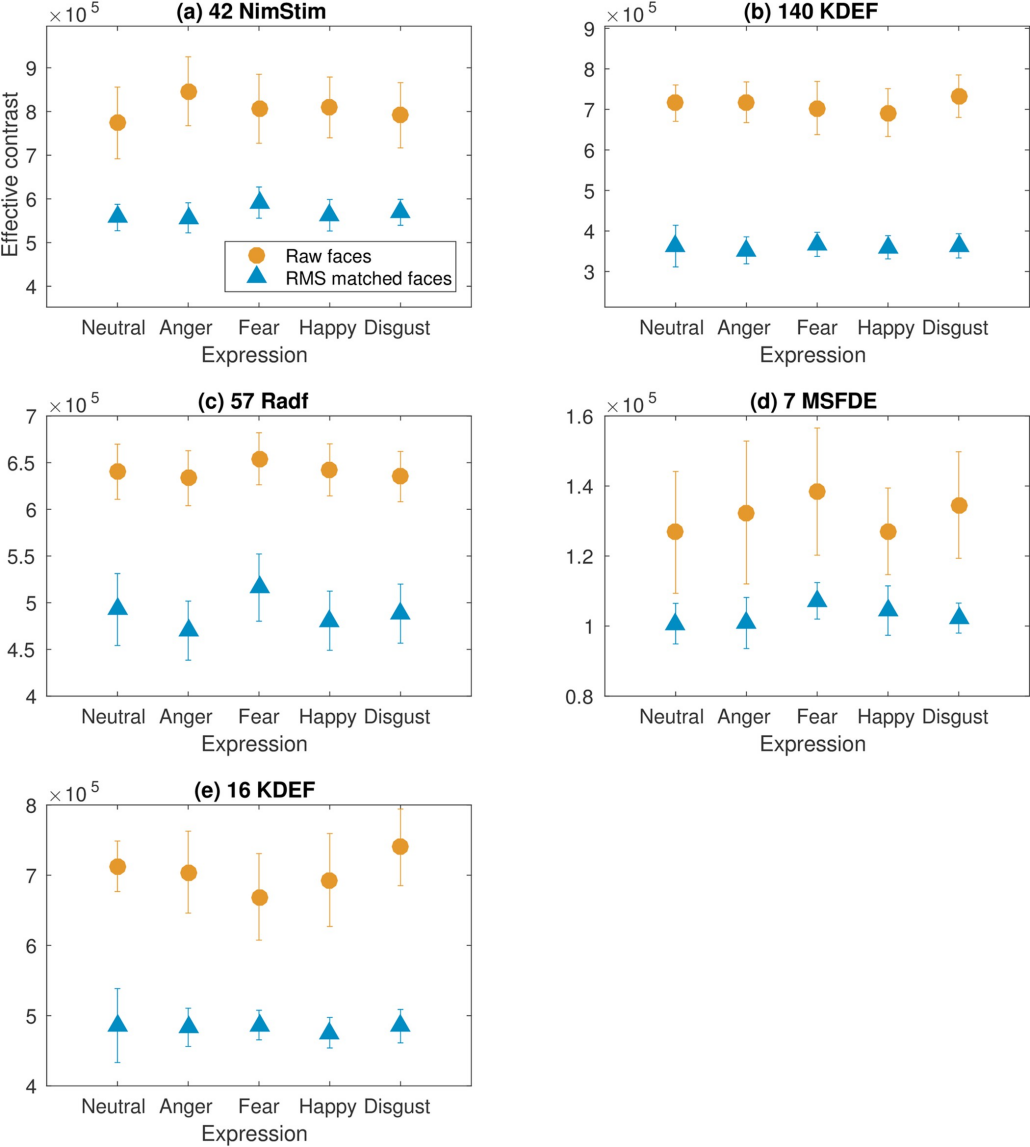
Expression-related differences in effective contrast across face image databases. Effective contrast for neutral faces, and anger, fear, happiness and disgust expressions, measured for raw faces (circle data) and the same faces normalised for RMS contrast (triangle data). Effective contrast measures were performed across 4 samples of face images, including the (A) NimStim, (B) KDEF, (C) Radboud, and (D) MSFDE, face sets employed by Hedger and colleagues [15], and for the 16 KDEF faces used in the present contrast sensitivity study (E). Error bars represent ±1 standard deviations.

As outlined by Hedger and colleagues [15] the overall estimate of effective contrast for each face image was obtained by summing contrast across spatial frequency after application of the contrast sensitivity model. All face images were analysed in two conditions: after they had been normalised for RMS contrast (according to Hedger and colleagues [15]), and also in their raw form, such that no contrast normalisation had taken place. In the RMS-matched analysis, the RMS contrast of each face was set to be equal to that of the image with the *lowest* contrast in each set. It is for this reason that the RMS-matched stimuli have an overall lower effective contrast. All face images depict forward-facing actors displaying one of 5 expressions (neutral, anger, fear, happy or disgust), cropped to include internal features only. The average effective contrast for each facial expression, compared across the 5 face image samples, including the experimental stimuli for the present contrast sensitivity study, is displayed in Fig 3.

*42 NimStim face images*: Effective contrast for neutral, angry, fearful, happy and disgust NimStim faces are shown in Fig 3A, and summarised in Table 1A. Sidak-corrected paired comparisons explored differences in effective contrast between fear expressions and neutral, anger, happy and disgust counterparts (alpha = 0.0127, accounting for 4 comparisons). When faces had been normalised for RMS contrast, NimStim fear expressions were significantly higher in effective contrast compared to all other expressions, including neutral faces. This finding is consistent with that observed by Hedger and colleagues [15], whereby RMS-normalised fear expressions were found to be significantly higher in effective contrast compared to neutral expressions. Alternatively, when faces were not normalised for RMS contrast, NimStim fear expressions were significantly higher in effective contrast compared to neutral faces, and lower in effective contrast compared to angry faces. No other significant differences were observed.

**Table 1:** Effective contrast compared between fear and counterpart expressions.

(a) 42 NimStim faces				
**Not normalised**	t	df	CI	Sig
Fear-neutral	3.97	41	15887.84, 48742.20	< .001
Fear-anger	-3.50	41	-3337.05, -16987.44	.001
Fear-happy	-.36	41	-21229.94, 14786.41	.72
Fear-disgust	1.27	41	-8667.98, 38236.24	.21
**RMS normalised**	t	df	CI	Sig
Fear-neutral	9.75	41	27128.97, 41299.98	< .001
Fear-anger	6.58	41	24173.68, 45546.27	< .001
Fear-happy	7.79	41	21670.22, 36397.82	< .001
Fear-disgust	5.62	41	14337.81, 30382.54	< .001
(b) 140 KDEF faces				
**Not normalised**	t	df	CI	Sig
Fear-neutral	-2.40	139	-21956.07, -2130.43	.018
Fear-anger	-2.47	139	-25622.43, -2854.71	.015
Fear-happy	1.81	139	-1011.76, 23548.61	.075
Fear-disgust	-4.96	139	-40878.60, -17581.18	< .001
**RMS normalised**	t	df	CI	Sig
Fear-neutral	.96	139	-4403.07, 12759.73	.33
Fear-anger	4.96	139	8864.86, 20593.09	< .001
Fear-happy	2.49	139	1473.99, 12835.65	.014
Fear-disgust	1.27	139	-1958.42, 8989.18	.20
(c) 57 Radboud faces				
**Not normalised**	t	df	CI	Sig
Fear-neutral	6.05	56	9302.71, 18501.62	< .001
Fear-anger	8.01	56	15594.01, 25979.80	< .001
Fear-happy	3.92	56	5821.49, 17985.59	< .001
Fear-disgust	9.00	56	14834.82, 23323.09	< .001
**RMS normalised**	t	df	CI	Sig
Fear-neutral	10.18	56	18898.93, 28150.01	< .001
Fear-anger	18.90	56	41225.73, 50999.21	< .001
Fear-happy	14.01	56	30423.72, 40576.68	< .001
Fear-disgust	12.10	56	23278.46, 32513.48	< .001
(d) 7 Montreal (MSFDE) faces				
**Not normalised**	t	df	CI	Sig
Fear-neutral	1.95	6	-2924.24, 26224.08	.09
Fear-anger	.60	6	-18156.74, 30104.36	.56
Fear-happy	1.78	6	-4255.20, 26954.75	.12
Fear-disgust	.73	6	-8982.68, 16669.87	.49
**RMS normalised**	t	df	CI	Sig
Fear-neutral	4.65	6	3087.98, 9943.33	.003
Fear-anger	2.82	6	843.11, 11834.65	.03
Fear-happy	1.82	6	-955.55, 6513.98	.18
Fear-disgust	2.67	6	424.47, 9425.20	.037
(e) 16 KDEF faces				
**Not normalised**	t	df	CI	Sig
Fear-neutral	-3.49	15	-70194.13, -16964.54	.003
Fear-anger	-1.86	15	-75544.43, 5119.73	.08
Fear-happy	-1.59	15	-56093.38, 8083.37	.13
Fear-disgust	-3.65	15	-111632.35, -29367.94	.002
**RMS normalised**	t	df	CI	Sig
Fear-neutral	.05	15	-29304.24, 30754.92	.96
Fear-anger	.43	15	-12714.96, 19164.70	.67
Fear-happy	2.53	15	1753.64, 20077.86	.02
Fear-disgust	.27	15	-10552.70, 13706.44	.78

Effective contrast compared between fear and counterpart expressions. Sidak- corrected comparisons (a = 0.0127, accounting for 4 comparisons) are made between raw, and thus not normalised faces, and also when they are matched for RMS contrast. Measures are performed for 4 databases (a-d), and experimental stimuli used in the present behavioural study (e).

**Fig 4:**
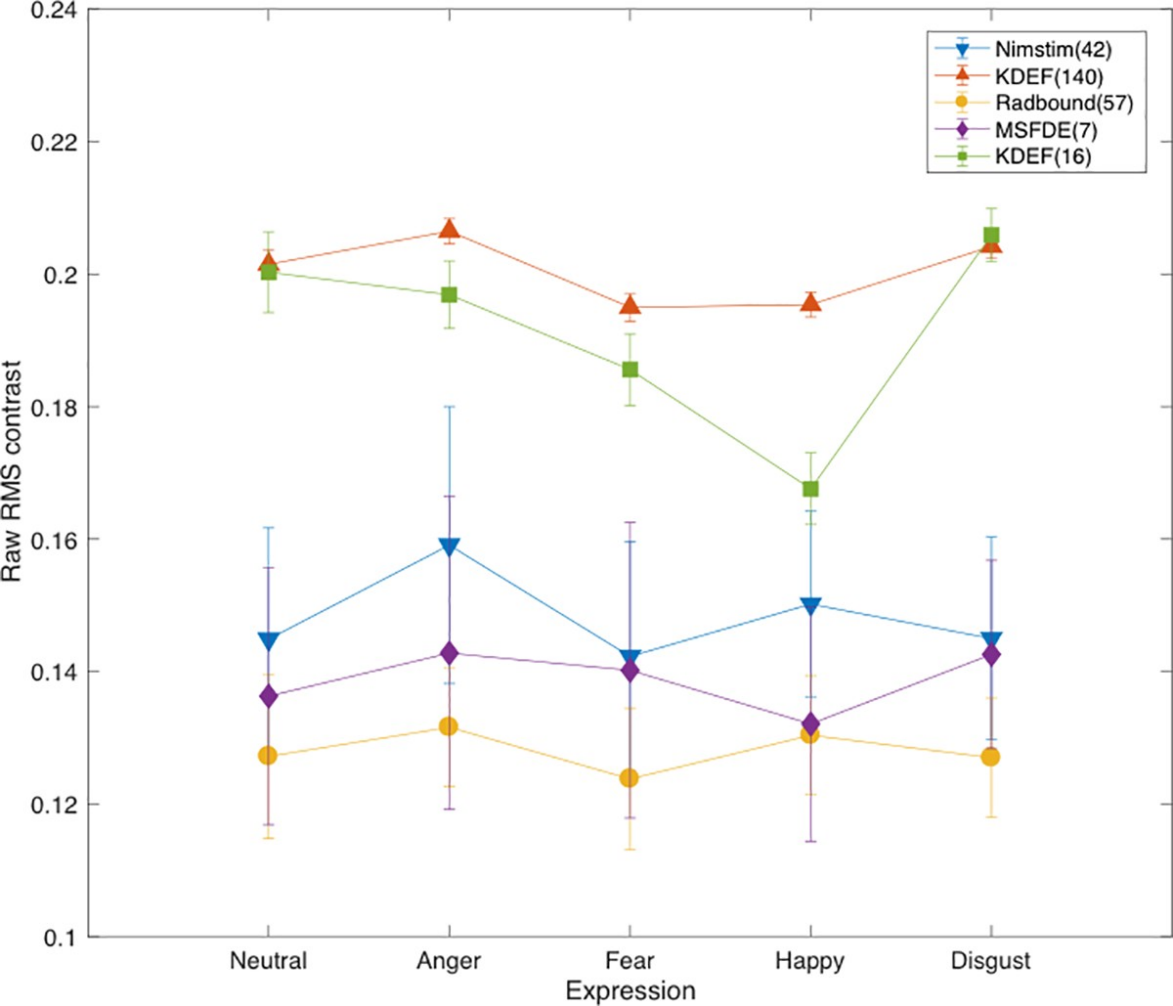
Expression-related differences in raw RMS contrast across face image databases. RMS contrast for face expressions before faces are subjected to contrast normalisation i.e. when kept in raw format. RMS contrast for 5 expressions is measured across the 5-database face samples used to calculate faces’ effective contrast. Error bars represent ±1 standard deviations.

For 42 raw (not normalised) NimStim faces, RMS contrast was calculated for the 5 face expressions to explore how natural differences in physical contrast compare with expression-related differences in effective contrast, and in particular, whether this is influenced by contrast normalisation. Sidak-corrected comparisons compared RMS contrast between fear expressions and each of their face counterparts, including neutral faces (alpha = 0.0127). Fearful NimStim faces naturally possessed significantly less RMS contrast compared to angry and happy expressions. No other significant differences were observed. These data are illustrated in Fig 4, and summarised in Table 2A.

**Table 2:** Differences in raw RMS contrast between raw fear expressions and 4 emotion counterparts.

Raw RMS:				
(a) 42 NimStim				
	t	df	CI	Sig
Fear-neutral	-1.27	41	-.006, .001	.21
Fear-anger	-5.46	41	-.023, -.010	< .001
Fear-happy	-4.09	41	-.011, -.003	< .001
Fear-disgust	-1.06	41	-.007, .002	.29
(b) 140 KDEF				
	t	df	CI	Sig
Fear-neutral	-2.67	139	-.011, -.001	.008
Fear-anger	-5.09	139	-.016, -.007	< .001
Fear-happy	-.19	139	-.005, .004	.85
Fear-disgust	-4.27	139	-.013, -.004	< .001
(c) 57 Radboud				
	t	df	CI	Sig
Fear-neutral	-4.37	56	-.005, -.001	< .001
Fear-anger	-10.24	56	-.009, -.006	< .001
Fear-happy	-7.05	56	-.008, -.004	< .001
Fear-disgust	-4.55	56	-.004, -.001	< .001
(d) 7 Montreal (MSFDE)				
	t	df	CI	Sig
Fear-neutral	.70	6	-.009, .017	.50
Fear-anger	-.26	6	-.025, .020	.79
Fear-happy	1.17	6	-.001, .025	.28
Fear-disgust	-.45	6	-.015, .010	.66
(e) 16 KDEF				
	t	df	CI	Sig
Fear-neutral	-2.42	15	-.027, -.001	.02
Fear-anger	-1.56	15	-.026, .004	.13
Fear-happy	-2.15	15	-.022, .000	.04
Fear-disgust	-3.46	15	-.032, -.007	.003

Differences between RMS contrast in raw fear expressions and 4 emotion counter- parts (a = 0.0127). Fear comparisons are measured across all 4 databases (a-d), and also for the experimental stimuli used in the present contrast sensitivity study (e).

*140 KDEF face images*: Effective contrast for neutral, angry, fearful, happy and disgusted KDEF faces are shown in Fig 3B, and summarised in Table 1B. Sidak-corrected paired comparisons explored differences in effective contrast between fear expressions and neutral, anger, happy and disgust counterparts (alpha = 0.0127). For KDEF faces normalised for RMS contrast, fear expressions are significantly higher in effective contrast compared to angry faces. Alternatively, when the same KDEF faces are not normalised for RMS contrast, fear expressions are significantly lower in effective contrast compared to disgust expressions. No other significant differences were observed.

For 140 raw (not normalised) KDEF faces, naturally-occurring and expression-related differences in RMS contrast were explored using Sidak-corrected comparisons (alpha = 0.0127). Fearful KDEF faces naturally contain significantly less RMS contrast compared to neutral, angry and disgust expressions. No other significant differences were observed. These data are illustrated in Fig 4, and summarised in Table 2B.

*57 Radboud face images*: Effective contrast for neutral, angry, fearful, happy and disgust Radboud faces are shown in Fig 3C, and summarised in Table 1C. Sidak-corrected paired comparisons explored differences in effective contrast between fear expressions and neutral, anger, happy and disgust counterparts (alpha = 0.0127). For Radboud face images normalised for RMS contrast, fear expressions are significantly higher in effective contrast compared to all other expressions, including neutral. Alternatively, when the same Radboud faces are not normalised for contrast, the same effect is true; raw fear expressions are significantly higher in effective contrast compared to all other expressions, including neutral. These findings in particular require further discussion, presented in the following section.

For 57 raw Radboud faces, naturally-occurring and expression-related differences in RMS contrast were explored using Sidak-corrected comparisons (alpha = 0.0127). Fearful Radboud faces naturally contained significantly less RMS contrast compared to all other face expressions. These data are illustrated in Fig 4, and summarised in Table 2C.

*7 Montreal (MSFDE) face images*: Effective contrast for neutral, angry, fearful, happy and disgust Montreal faces are shown in Fig 3D, and summarised in Table 1D. Sidak-corrected paired comparisons explored differences in effective contrast between fear expressions and neutral, anger, happy and disgust counterparts (alpha = 0.0127). For Montreal faces that are normalised for RMS contrast, fear expressions are significantly higher in effective contrast compared to neutral faces. Alternatively, when the same Montreal faces are not normalised for RMS contrast, effective contrast in fear expressions does not differ significantly compared to any other face. No other significant differences were observed.

For 7 raw Montreal faces, naturally-occurring and expression-related differences in RMS contrast were explored using Sidak-corrected comparisons (alpha = 0.0127). Fearful Montreal faces do not naturally differ in terms of RMS contrast compared to any other face expression, including neutral faces. These data are illustrated in Fig 4, and summarised in Table 2D.

*16 KDEF face images (experimental stimuli)*: Effective contrast for the experimental face stimuli used in our contrast sensitivity study are shown in Fig 3E, and summarised in Table 1E. Sidak-corrected paired comparisons explored differences in effective contrast between fear expressions and neutral, anger, happy and disgust counterparts (alpha = 0.0127). For the 16 KDEF (experimental stimuli) that were normalised for RMS contrast, no differences in effective contrast were observed between fear and any other expression, including neutral. Alternatively, when the same faces were not normalised for RMS contrast, fear expressions are significantly lower in effective contrast compared to both neutral and disgust faces. No other significant differences were observed.

For the 16 raw KDEF faces used in the present contrast sensitivity study, naturally-occurring and expression-related differences in RMS contrast were explored using Sidak-corrected comparisons (alpha = 0.0127). Experimental fearful expressions were lower in RMS contrast compared to disgust. No other significant differences were observed. These data are illustrated in Fig 4, and summarised in Table 2E.

Together, data from the present contrast sensitivity study showed that visual contrast thresholds are not influenced by differences between images of facial expressions. Namely, fearful expressions portrayed by face images did not enhance observers’ contrast sensitivity, as was predicted by findings from Hedger, Adams and Garner [15]. Fearful expressions, according to image analyses by Hedger, Adams and Garner [15] are higher in effective contrast, and thus well-tuned to contrast processing. This proposal was driven by data from image analyses measuring differences in effective contrast between fear and neutral face images that had been normalised for RMS contrast. The stimuli used in the present study were raw face images that were not normalised for physical contrast in any way. We replicate measures of effective contrast used by Hedger, Adams and Garner [15] to establish the extent that CSF advantages exclusive to fear expressions may be driven to some extent by effects of contrast normalisation on the effective contrast of faces. A general, but not always consistent, trend across the present image analyses is that greater effective contrast in fear expressions is influenced by whether or not face images are first normalised for RMS contrast. This was the case for the KDEF database; a set of facial stimuli used as both the experimental stimuli in the present CSF study, and that which was included in image analyses conducted by Hedger and colleagues [15]. Importantly, although there is a fair pattern of effects that favour effective contrast in RMS normalised compared to raw fear expressions, it is important to note that this was not true across analyses for all face databases, including Montreal and Radboud face samples.

## Experiment 2: Visibility of facial expressions under backward-masking conditions

Hedger and colleagues [15] show that the effective contrast of fear, angry, happy and neutral faces is a predictor of expressions’ visibility under visual suppression conditions (namely, using a continuous flash suppression paradigm). Here, fear expressions were associated with both greater amounts of effective contrast, and overall better detection [15]. To directly address whether the findings from Hedger, Adams and Garner [15] reflect an unintended effect of contrast normalisation on the salience of fearful expressions, Experiment 2 conducts a replication of their backward masking paradigm. Their study measured observers’ detection accuracy for backward-masked neutral, fearful, angry and happy expressions: face stimuli that had been normalised for RMS contrast. The findings showed that the degree of effective contrast belonging to such fearful expressions was a significant predictor of their detectability under backward masking conditions. Findings from Experiment 1 and image analyses above suggests the possibility that routinely normalising facial stimuli for RMS contrast can increase their effective contrast in a way that might enhance their perceived salience. Experiment 2 addresses the question of whether the association between the advantage to detect backward-masked fearful expressions and their effective contrast content rely on these facial expressions having first been normalised for RMS contrast. The experimental design of Experiment 2 is almost identical to the design used by Hedger and colleagues [15], with the differences being our use of KDEF faces to maintain consistency with Experiment 1, and the addition of two conditions for the contrast in faces: we include a condition for face images that have not been normalised for physical contrast in any way, such that they are presented in their raw contrast format, and a second condition for face images that are normalised for RMS contrast. Contrast normalisation of face stimuli followed the stimulus specifications used by Hedger and colleagues [15]. Findings from Experiment 1 suggest that fearful expressions are not naturally higher in effective contrast -suggested by Hedger and colleagues [15] as a possible mechanism of a fearful face bias- rather, that this effect may be facilitated when face stimuli are first normalised for RMS contrast. It may be that the process of contrast normalisation can enhance faces’ salience and therefore the effects of emotional expression on detection in a backward-masking paradigm. If so, we expect these effects to be reduced or eliminated when stimuli are not matched for RMS contrast.

## Materials and methods

### Participants

Fifteen participants (12 women, 3 men) took part in the study. Participants’ age ranged between 19 and 26 years old. All participants were informed of the nature of the study and provided informed consent prior to the study beginning. The University of Essex Ethics Committee approved the employed experimental procedures. All participated in the experiment as part of a credited research module assessment, or in exchange for monetary reward. All participants had normal to corrected vision.

### Stimuli and apparatus

Stimulus specifications matched those used in Experiment 1, with the exception that only 4 KDEF [18] actors of the original 16 were selected for Experiment 2 (image analyses). All individual faces were presented in their normal, upright form, and in a manipulated format. Manipulated versions of faces, following the procedure of Hedger, Adams and Garner [15], were rotated by 180° and subjected to a reversal of luminance polarity. Similar to Fourier phase scrambling face images, manipulating facial stimuli in this way disrupts their configural content, while preserving low-level image properties including contrast and spatial frequency content [7, 15]. Raw face stimuli included faces that had not been normalised for physical contrast in any way, such that they contained natural variability in terms of their contrast content. RMS normalised versions of raw faces were included as a second contrast condition, where each face image was assigned the average RMS value generated from all face stimuli; consistent with stimuli used by Hedger, Adams and Garner [15].

Forward and Backward masks were formed of random noise patterns, with a random phase spectrum. The Fourier amplitude spectrum of masks was matched to the average of all facial stimuli. Phase scrambled non-target stimuli consisted of Fourier phase scrambled versions of each target face image, for which the assigned Fourier amplitude value also matched the average across all facial stimuli. This was to ensure that possible salience differences between non-target phase scrambled and masking stimuli did not inadvertently influence the perceived salience of target faces.

Stimuli were presented using a VIEPIXX 3D monitor (52 cm X 29cm), with a resolution of 1920 X 1080 pixels, a refresh rate of 120Hz, and an average luminance of 50 cdm^-2^. Each pixel subtended 1.43 arc min. Stimuli were presented at 10 bit resolution, and viewed from a distance of 80cm. The size of each face image was 5.8° face-width. Participant responses were recorded using the RESPONSEPixx response box. Stimuli were generated and presented using MATLAB and the Psychophysics Tool box extensions [24–26].

### Procedure

The beginning of a trial started with a central fixation cross, presented for 500ms. Next, two forward masks were presented to the left and right of centre for 200ms, followed by both a target face image and a non-target Fourier phase scrambled face. Target images were presented for one of 8 stimulus durations (16.6, 24.9, 33.2, 49.8, 66.4, 83.0, 99.6 and 116.2ms). Next, two backward masks were presented for 200ms. In a 2AFC task, participants were instructed to press a left or right button, corresponding to the side of the display that a face appeared. The display remained blank until the point at which participants indicated whether the target had appeared to the left or right of centre. Participant responses initiated the onset of the next trial. Overall, there were 2048 trials: 4 (expressions) x 2 (manipulation conditions) x 2 (contrast conditions) x 8 (durations) x 4 (actors) x 4 (repetitions). Trials were spread across 4 blocks containing 512 trials. Two blocks consisted of raw, non-normalised face images (raw contrast condition), and two blocks consisted of RMS contrast normalised face images. Block order was randomised between participants. Responses were combined across actors to give 16 repetitions for each combination of the other parameters.

## Results

**Fig 5:**
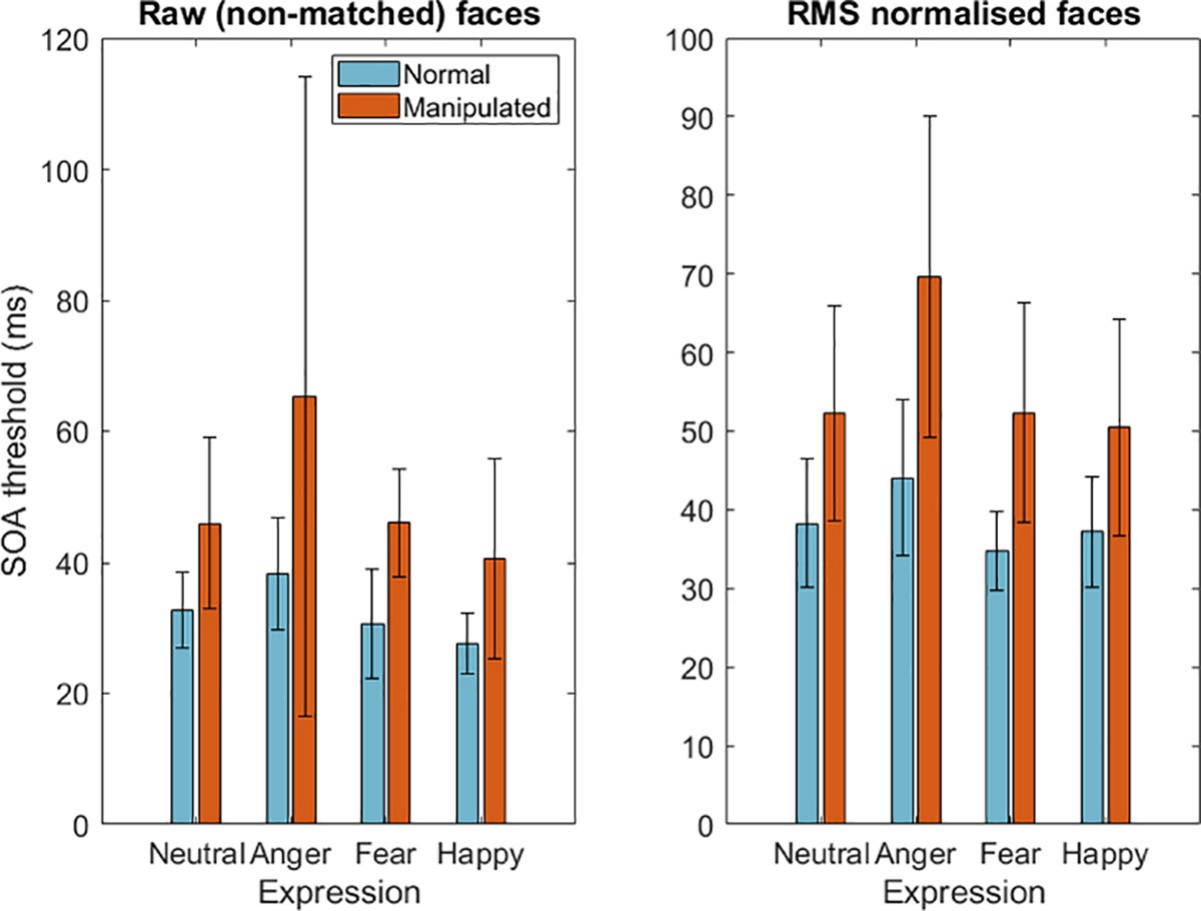
Stimulus Onset Asynchrony (SOA) thresholds for expressions. Left figure displays thresholds for facial stimuli when they are presented in their raw, non-contrast normalised format. Right figure displays thresholds for facial stimuli that were normalised for their RMS contrast content. Error bars represent ±1 standard deviations.

For each condition (contrast, image manipulation and emotional expression) a psychometric function was produced for each individual, indicating the proportion of correct responses as a function of the SOA. From these, a 75 percent correct threshold was calculated. Fig 5 plots the mean thresholds for each expression, for manipulated and unmanipulated stimuli. Thresholds are plotted separately for RMS-contrast matched and unmatched stimuli. These data were analysed using a 2 (contrast match) x 2 (image manipulation) x 4 (facial expression) repeated measures analysis of variance. There was a significant main effect of contrast-matching (*F*(1,14) = 22.59, *p* < .001, ηp ^2^ = .617). Stimuli could be detected at shorter durations when they were not normalised for contrast. This reflects the reduction in contrast introduced in the RMS matching procedure. Unmanipulated faces could be detected at significantly shorter durations compared their manipulated versions (*F*(1,14) = 30.74, *p* < .001, ηp ^2^ = .687). Stimulus detection was also significantly affected by emotional expression (*F*(3,42) = 15.87, *p* < .001, ηp ^2^ = .531), explored below. There were no significant two-way or three-way interactions.

The effect of expression was assessed in detail using pairwise corrections with Sidak corrections for multiple comparisons (alpha = 0.0085, according to 6 comparisons). These were performed separately for the RMS-contrast matched and unmatched conditions. For both RMS normalised and raw faces, SOA thresholds were significantly longer for angry compared to fearful faces. This effect was preserved for manipulated versions of angry faces, but only when they had been RMS normalised. No other significant differences were observed. These data are presented in Table 3.

**Table 3:** Differences in stimulus detection between facial expressions in a backward masking paradigm.

(a) SOAs RMS normalised faces				
	t	df	CI	Sig
Fear-neutral	-2.29	14	-6.86, -.23	.03
Fear-anger	-4.12	14	-14.17, -4.47	.001
Fear-happy	-1.31	14	-6.35, 1.53	.212
*Manipulated faces*				
Fear-neutral	.02	14	-6.800, 6.93	.98
Fear-anger	-3.95	14	-26.58, -7.89	.001
Fear-happy	.51	14	-5.97, 9.76	.61
(b) SOAs Raw (not normalised) faces				
	t	df	CI	Sig
Fear-neutral	-1.03	14	-6.44, 2.24	.31
Fear-anger	-3.87	14	-11.96, -3.44	.002
Fear-happy	1.63	14	-.94, 7.04	.124
*Manipulated faces*				
Fear-neutral	.04	14	-4.40, 4.59	.96
Fear-anger	-1.73	14	-42.97, 4.52	.10
Fear-happy	2.18	14	.09, 10.87	.04

Differences in detection thresholds under backward masking conditions: compared between fear and counterpart faces (a = 0.0085).

While our results do not provide evidence of a threat bias (better performance for fearful faces), the robustly poorer performance for angry faces is a consistent finding across studies [15]. As noted, this effect in itself conflicts with the idea that threatening stimuli are more readily detected [15]. The critical finding for the current experiment is that the effects of emotional expression on detection responses were *not* affected by the presence or absence of matching for RMS contrast. This means that these effects are unlikely to be an artefact of contrast normalisation.

## Discussion

A widely accepted view in the threat bias literature is that fearful face expressions possess a special status in the human visual system, due to their low level image content [1, 3–4, 7, 29]. Hedger, Adams and Garner [15] recently showed that the visibility, or salience, associated with fear expressions is predicted by their effective contrast content; the extent that the Fourier amplitude of fear expressions, compared to neutral faces, exploits the contrast sensitivity function. In the present study, we conducted a traditional contrast sensitivity task to test whether higher effective contrast purported for fear expressions is associated with lower visual contrast thresholds at the behavioural level. We measured contrast sensitivity for facial stimuli of 5 raw face expressions. No expression-related differences were observed across visual thresholds, in contrast with our predictions based on data from Hedger Adams and Garner [15]. Specifically, a decrease in visual thresholds for fearful expressions was not observed. Greater effective contrast unique to fear expressions (when compared to neutral faces) was observed by Hedger and colleagues [15], but only for face images that had been normalised for RMS contrast. In order to investigate whether the use of contrast normalisation by Hedger, Adams and Garner [15] may have driven effective contrast effects that in its absence were not replicated by our contrast sensitivity study, we repeated calculations of effective contrast using the same procedure employed by Hedger, Adams and Garner [15]. Effective contrast was calculated for images of face expressions both when they were normalised for RMS contrast, as was performed by Hedger, Adams and Garner [15], but also when the same faces had not been normalised for physical contrast. These analyses were performed for the NimStim, KDEF, Montreal (MSFDE) and Radboud face sets used by Hedger and colleagues [15], and also for the 16 KDEF face images used as the experimental stimuli in the present contrast sensitivity study. Importantly, our findings replicate those of Hedger, Adams and Garner [15], showing that fear expressions normalised for RMS contrast are often significantly higher in effective contrast than neutral counterparts. We extend this finding to show that this is also true when fearful faces are compared to other face expressions. This advantage was observed for NimStim, KDEF and Radboud face databases. However, when the same faces were analysed in their raw form (i.e. when they were not normalised for physical contrast), this effect of fear diminishes for NimStim and KDEF face databases. These findings indicate that the process of normalising face stimuli, to some extent, can increase the effective contrast in fearful face expressions, where naturally (not normalised) such faces tend not to differ in effective contrast compared to other facial expressions, or indeed are more likely to be lower in effective contrast. An important finding to discuss here is the absence of this contrast normalisation effect for face images taken from the Radboud face database. We observed that Radboud fear expressions normalised for contrast were significantly higher in effective contrast compared to neutral faces, as well as other expressions; an effect that is consistent with that observed by Hedger and colleagues [15]. However, this effect did not diminish when images were not normalised for contrast; an effect that was not found for other face samples. Radboud face images were included in the present study on the basis that they were included in the original study by Hedger and colleagues [15]. Details of the image processing used to create and standardise these actor photographs includes white-balance correction [19]. This process adjusts raw image data in order to remove certain unrealistic and biased appearances, such as those incurred under different lightning conditions [30, 31]. It is important to note that database production information for KDEF and NimStim face sets do not refer to any image processing related to white-balance correction, or contrast normalisation [32]. No information about image processing is provided for the Montreal image database. It may therefore be that contrast and luminance information in ‘raw’ Radboud face images had already been subjected to some degree of normalisation, or standardisation, when they were created.

Given that the normalisation for RMS contrast routinely applied tends to increase differences in effective contrast, we performed a backward-masking study with normalised and non-normalised facial stimuli. Hedger and colleagues [15], using the same paradigm, found that fearful faces required shorter stimulus exposures (SOAs) compared to neutral and angry faces under conditions where face images had been normalised for RMS contrast. Findings from the present backward-masking study also demonstrate longer SOA detection thresholds associated with angry facial expressions, but importantly, that effects of emotional expression do not rely on these faces having been normalised for RMS contrast. Our findings show that normalising facial stimuli for RMS contrast has an overall effect on faces’ detectability. In a recent study, Menzel, Redies and Hayn-Leichsenring [33] found that the process of normalising face stimuli in terms of their low-level image properties–including their RMS contrast and brightness- had an inhibitory effect on observers’ ability to perceptually match facial expressions, compared to when naturally occurring differences in such image properties were left uninfluenced by contrast normalisation. Findings from the same study [33] also showed that angry facial expressions are naturally higher in RMS contrast, consistent with findings from our image analyses. Importantly, findings from the present study support the existing literature [33] that suggests that facial expressions contain naturally occurring differences in terms of their low-level image properties, and that these differences appear to play an important role for efficient detection of facial stimuli. The process of normalising facial stimuli for their RMS contrast does artificially alter their resulting effective contrast, but at the behavioural level, this inadvertent increase in effective contrast for fear expressions does not account for the fear detection advantage under conditions of backward-masking. This shows that these effects are not simply an artefact of contrast normalisation.

In sum, the present study performed a traditional contrast sensitivity task to address the proposal that fearful faces exploit the contrast sensitivity function, and as a result undergoes efficient visual processing [15]. Together, these findings suggest that contrast normalisation–a standard procedure in psychophysical studies- significantly influences the physical composition of face stimuli in a way that might be expected to influence their perceived salience under both experimental and neurophysiological conditions. However, when this is considered, significant differences between expressions remain in both effective contrast and detectability.
